# Effects of Chemically-Modified Polypyridyl Ligands on the Structural and Redox Properties of Tricarbonylmanganese(I) Complexes

**DOI:** 10.3390/molecules25245921

**Published:** 2020-12-14

**Authors:** Takatoshi Kanno, Tsugiko Takase, Dai Oyama

**Affiliations:** 1 Graduate School of Science and Engineering, Fukushima University, 1 Kanayagawa, Fukushima 960-1296, Japan; s1970013@ipc.fukushima-u.ac.jp; 2 Department of Natural Sciences and Informatics, Fukushima University, 1 Kanayagawa, Fukushima 960-1296, Japan; ttakase@sss.fukushima-u.ac.jp

**Keywords:** manganese, crystal structure, redox reaction, phenanthroline, bipyridine, carbonyl complex, quinone, catechol, methyl viologen, interconversion

## Abstract

Carbonyl complexes with manganese(I) as the central metal are very attractive catalysts. The introduction of redox-active ligands, such as quinones and methyl viologen analogs into these catalysts, would be expected to lead to superior catalyst performances, since they can function as excellent electron carriers. In this study, we synthesized four tricarbonylmanganese(I) complexes containing typical bidentate polypyridyl ligands, including 1,10-phenanthroline (phen) and 2,2′-bipyridine (bpy) frameworks bound to redox-active *ortho*-quinone/catechol or methyl viologen-like units. The molecular structures of the resulting complexes were determined by X-ray crystallography to clarify their steric features. As expected from the infrared (IR) data, three CO ligands for each complex were coordinated in the *facial* configuration around the central manganese(I) atom. Additionally, the structural parameters were found to differ significantly between the quinone/catechol units. Electrochemical analysis revealed some differences between them and their reference complexes, namely [MnBr(CO)_3_(phen)] and [MnBr(CO)_3_(bpy)]. Notably, interconversions induced by two-electron/two-proton transfers between the quinone and catechol units were observed in the phenanthroline-based complexes. This work indicated that the structural and redox properties in tricarbonylmanganese(I) complexes were significantly affected by chemically modified polypyridyl ligands. A better understanding of structures and redox behaviors of the present compounds would facilitate the design of new manganese complexes with enhanced properties.

Academic Editor: Rudy J. Richardson

## 1. Introduction

Due to the fact that manganese, a Group 7 element, is located in the center of the *d*-block elements on the periodic table, it has the widest oxidation number of the first-row transition metals (i.e., +7 state). As a result of its unique properties, manganese compounds such as KMnO_4_ and MnO_2_ have long been used as versatile oxidants and oxidation catalysts. Manganese is also present in the oxygen-evolving complex (OEC) that takes part in photosynthesis, where it plays an important role in catalyzing the oxidation of water [1].

**Scheme 1:**
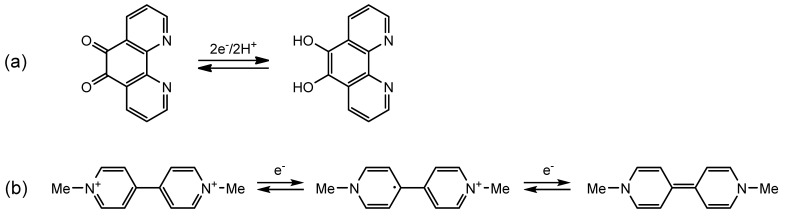
(**a**) Direct reversible 2e^−^/2H^+^ process of dpq, and (**b**) successive reversible 2e^−^ process of methyl viologen.

On the other hand, manganese compounds containing π-acidic ligands, such as carbonyl (CO), generally exhibit lower valences. In this type of compound, since manganese(I) is overwhelmingly abundant compared to similar *d*^6^ complexes of other noble metals (e.g., Re(I) or Ru(II) [2]); the replacement of these noble metals by first-row transition metals is of particular importance from an applied and industrial standpoint [3]. In manganese(I) carbonyl complexes containing a typical bidentate polypyridyl ligand (N-N) such as 1,10-phenanthroline (phen) or 2,2′-bipyridine (bpy) of the type [MnL(CO)_3_(N-N)]*^n^*^+^ (L = monodentate ligand, *n* = 0 or 1), much attention has been paid to the catalytic reactions based on the electrochemistry and photochemistry of the complex [4,5,6,7,8,9,10,11,12]. In this context, the synthesis of a manganese(I) carbonyl complex containing 1,10-phenanthroline-5,6-dione (dpq) as a ligand in which a redox-active quinone unit is introduced into the phenanthroline flamework ([MnBr(CO)_3_(dpq)]: **Mn-dpq**) has recently been reported, as has the application of such a system in the catalytic reduction of CO_2_ [13]; however, the molecular structure of **Mn-dpq** has yet to be determined. In addition, although the quinone moiety reversibly generates its reduced form (i.e., catechol) by a two-electron/two-proton process (Scheme 1a), the corresponding catechol complex has not been prepared.

**Figure 1:**
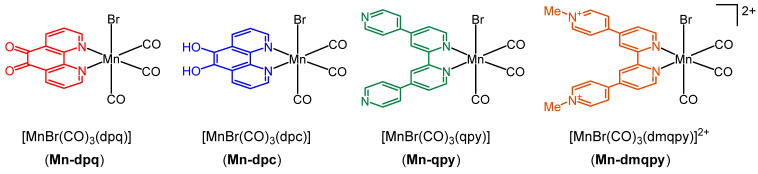
Chemical structures of the Mn(I) complexes presented in this study.

Thus, we herein describe the synthesis and molecular structure of the reduced compound [MnBr(CO)_3_(dpc)]: dpc = 5,6-dihydoxy-1,10-phenanthroline (**Mn-dpc**), as well as the molecular structure of the abovementioned **Mn-dpq** (Figure 1). In addition to a comparison of the structure and properties of both complexes, the redox-induced interconversion between these complexes is investigated in detail. Furthermore, we attempt the synthesis of a further manganese(I) system that contains 4,4′-substituted bipyridine units, which are representative of polypyridyl ligands; one is replaced with pyridine (4,4′:2′,2′’:4′’,4′’’-quaterpyridine; qpy), and the substituted pyridines are further quaternized to form the redox-active pyridinium compound (*N’’*,*N’’’*-dimethyl-4,4′:2′,2′*’*:4′*’*,4′’’-quaterpyridinium ion; dmqpy^2+^). In particular, since the latter ligand (dmqpy^2+^) is similar to methyl viologen, which functions as a multi-electron carrier (Scheme 1b) [14]; the introduction of the ligand into the manganese(I) complex would be expected to lead to an improvement in its reduction potential [15]. In addition to the phenanthroline-based manganese(I) complexes described above, the molecular structures and the redox properties of these bipyridine-based complexes (i.e., [MnBr(CO)_3_(qpy)]; **Mn-qpy** and [MnBr(CO)_3_(dmqpy)]^2+^; **Mn-dmqpy**) are also examined (Figure 1). Furthermore, the structures and redox properties of polypyridylmanganese(I) complexes containing these two different redox-active units are compared with their reference complexes ([MnBr(CO)_3_(phen)]; **Mn-phen** and [MnBr(CO)_3_(bpy)]; **Mn-bpy**, respectively), and the diversification of molecules caused by the introduction of redox-active ligands is evaluated.

## 2. Results and Discussion

### 2.1. Synthesis and Spectroscopic Characterization of the Tricarbonylmanganese Complexes

**Scheme 2:**
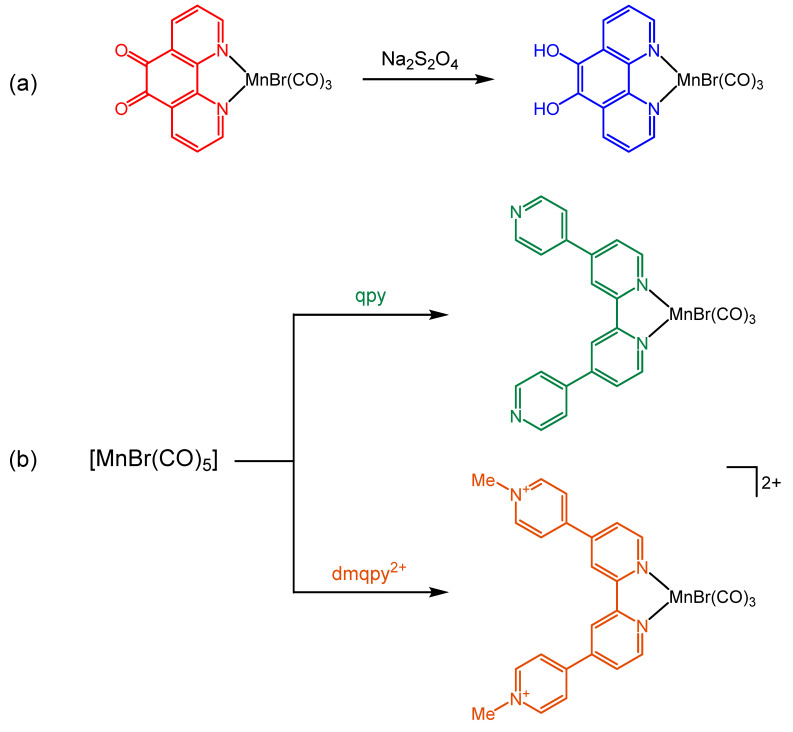
Synthetic routes to the Mn(I) complexes: (**a**) phenanthroline-based complexes and (**b**) bipyridine-based complexes.

Chemical reduction of the metal-free dpq to dpc was previously reported through the use of hydrazine, sodium borohydride (NaBH_4_), dithiooxamide, and sodium dithionite (Na_2_S_2_O_4_) [16,17,18,19,20]. In contrast, reduction of the dpq ligand in metal complexes was achieved during the formation of a heterodimer between the {Ru(dpq)}^2+^ site and M^0^ compounds (M = Pd or Pt) [21]. In this study, when the reduced catechol molecule (dpc) was reacted with [MnBr(CO)_5_], the coordinated dpc ligand was partially oxidized during the reaction to give a mixture of [MnBr(CO)_3_(dpq)] and [MnBr(CO)_3_(dpc)] under the heating conditions required for this reaction to take place. Therefore, the quinone-coordinated complex was initially prepared according to a previously reported procedure [13], and the corresponding catechol complex was subsequently obtained by chemical reduction at a low temperature (Scheme 2a). The conversion of quinone to catechol was confirmed by the absence of a C=O stretching band (1698 cm^−1^) at the quinone unit in the infrared (IR) spectrum. Meanwhile, no OH proton signal was observed in the ^1^H-NMR spectrum [22]. Since the catechol unit of the complex in solution is gradually oxidized by air to produce the corresponding quinone unit, the quinone form can be considered more stable in terms of this system (vide infra).

In the bipyridine-based complexes, on the other hand, qpy and dmqpy^2+^, which were substituted at the 4,4′-position of bpy with pyridine and pyridinium ions, respectively, were synthesized according to previously reported procedures [23,24], and then, each ligand was reacted with [MnBr(CO)_5_] to afford the corresponding 4,4′-substituted bipyridine complexes (Scheme 2b). In the elemental analysis of the isolated **Mn-dmqpy**, the calculated value was satisfactory when PF_6_^−^ was a counterion. However, when single crystals of this compound were prepared, single crystal X-ray structural and energy-dispersive X-ray (EDX) analyses suggested that both PF_6_^−^ and Br^−^ were mixed as counterions (results described later). Therefore, it is expected that bromide ions are also contained in a part of the isolated complex, and the PF_6_/Br salt present as a minor component is more likely to crystallize, leading to the selective precipitation of single crystals of the PF_6_/Br mixture. In addition, the quaternized unit was stably maintained during this complexation, since the methyl signal was observed at 4.44 ppm (^1^H) and 49.45 ppm (^13^C) in the NMR spectra of **Mn-dmqpy**.

**Figure 2:**
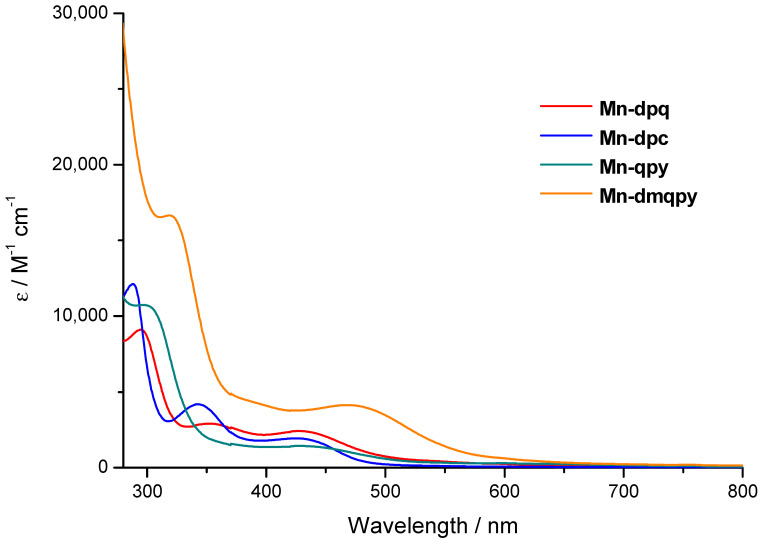
UV-visible spectra of the Mn(I) complexes (0.1-mM solutions in CH_3_CN).

In the IR measurements of the synthesized complexes, three CO-stretching bands attributed to the terminal carbonyl moieties were observed (Table 1). This suggests that the coordination geometries of all complexes are in the *facial* configuration, which is in agreement with the X-ray structural analyses results described below. In addition, the UV-visible spectra of acetonitrile solutions of the complexes showed several absorption bands in the visible region, which were attributed to metal-to-ligand charge transfer (MLCT) and/or halogen-to-ligand charge transfer (XLCT) (Figure 2 and Table 1) [13,25,26], and the phenanthroline-based complexes exhibited a red shift of ~20 nm compared to the reference **Mn-phen** [13]. Although the absorption in the visible region remained relatively unchanged upon reduction of the quinone unit to catechol, the extinction coefficient at the higher energy region (~290 nm) increased. Considering that the high energy absorption was assigned to intra-ligand π-π* transitions [13], this distinction indicates that dpq and dpc ligands exhibit different energy states despite their identical molecular frameworks. On the other hand, **Mn-qpy** was not significantly different from the reference **Mn-bpy** in the bipyridine-based complexes, and the absorption maximum of **Mn-dmqpy** was red-shifted by 40 nm, due to quaternization of the free pyridine units. Furthermore, since the absorbance in the visible region was also increased by approximately three times, it was apparent that introduction of the redox-active unit greatly affected the electronic states of the present complexes.

### 2.2. Structural Characterization of the Tricarbonylmanganese Complexes

Structural analyses were then performed on the four complexes synthesized in this work. As expected from the IR data, it was confirmed that the three CO ligands of all complexes were coordinated in the *facial* configuration around the central manganese(I) atom. The main bond parameters (Mn–C, Mn–N, Mn–Br, and C–O lengths and Mn–C–O angles) shown in Table 2 are similar to those reported in *fac*-[MnBr(CO)_3_(N-N)]-type complexes [4,25,26,27,28,29,30,31,32]. On the basis of these bond parameters, all CO ligands were concluded to exhibit typical triple-bond characteristics. Furthermore, the calculated density functional theory (DFT)-optimized structures of the complexes were found to be analogous to their experimentally determined structures (Table 2). For example, the Mn–N bond lengths were calculated to be 2.063–2.080 Å, which corresponds to the experimentally determined values of 2.0344(15)–2.0614(19) Å.

**Figure 3:**
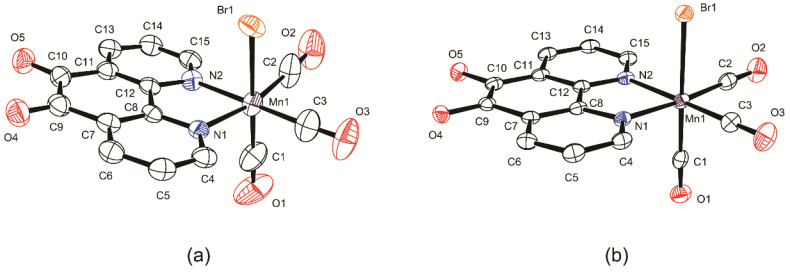
Molecular structures with atom labels and displacement ellipsoids for non-H atoms drawn at the 50% probability level. H atoms and the solvent molecule are omitted for clarity. (**a**) *fac*-[MnBr(CO)_3_(dpq)] and (**b**) *fac*-[MnBr(CO)_3_(dpc)].

**Figure 4:**
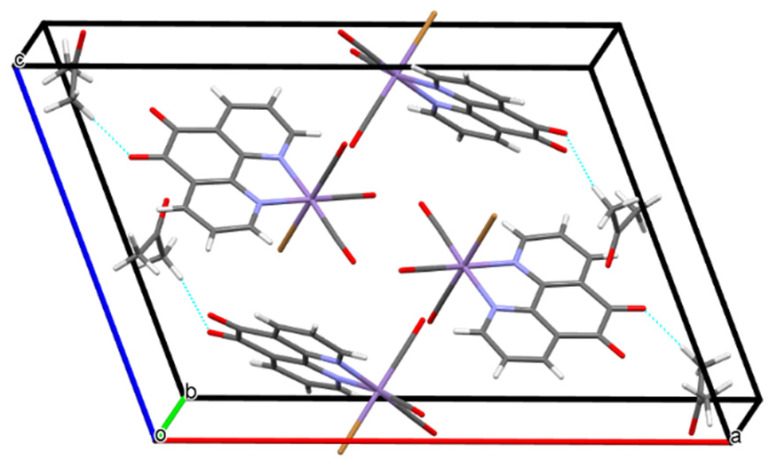
Crystal packing of [MnBr(CO)_3_(dpq)]·(CH_3_)_2_CO with C–H^…^O hydrogen bonds indicated by blue dashed lines.

As shown in Figure 3, although the coordination geometries of the two phenanthroline-based complexes are essentially the same, the structural parameters of the redox-active sites are quite different (Table 2). More specifically, the C–O bond lengths at the quinone unit in **Mn-dpq** are 1.212(6) and 1.220(7) Å; these smaller values are typical for C=O bonds. In contrast, the C–OH bond lengths of the catechol unit in **Mn-dpc** are 1.362(2) and 1.367(2) Å, which are within the range of C–O single-bond distances; these values are similar to those in the previously reported metal-free dpq and dpc, respectively [33,34]. Therefore, from a crystallographic point of view, it was confirmed that **Mn-dpq** and **Mn-dpc** possess quinone and catechol units, respectively. In addition, **Mn-dpq** possesses hydrogen bonds between the complex and the solvent molecules (acetone) (Figure 4 and Table S1), which may contribute to stabilization of the crystal. Meanwhile, intermolecular O–H^…^Br hydrogen bonds and a distinct π-π stacking (3.445(1) Å for the centroid-centroid distance between the central aromatic rings), as well as intramolecular hydrogen bonds between the two OH groups, were confirmed in **Mn-dpc**, given its tight dimer formation (Figure S1). These interactions perhaps correlate with the calculated density of the complexes.

**Figure 5:**
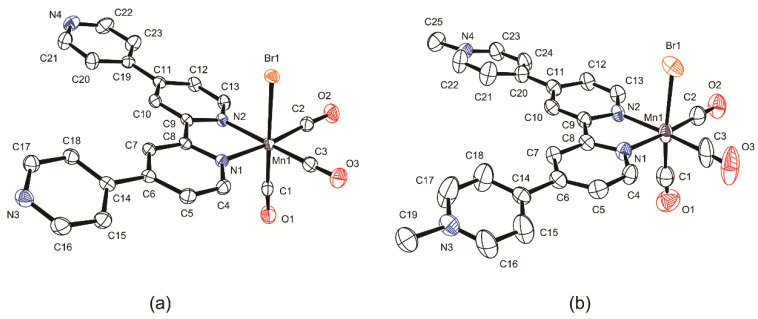
Molecular structures with atom labels and displacement ellipsoids for non-H atoms drawn at the 50% probability level. Counter-anions and H atoms are omitted for clarity. (**a**) *fac*-[MnBr(CO)_3_(qpy)] and (**b**) *fac*-[MnBr(CO)_3_(dmqpy)]^2+^.

**Figure 6:**
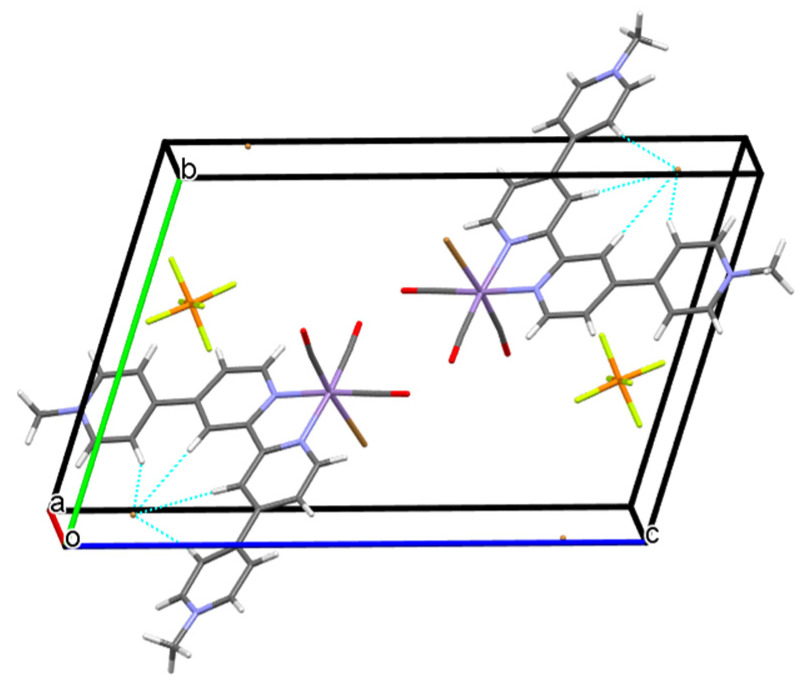
Crystal packing of [MnBr(CO)_3_(dmqpy)]Br(PF_6_) with the C–H^…^Br hydrogen bonds indicated by blue dashed lines.

The molecular structures of the bipyridine-based complexes are shown in Figure 5. More specifically, **Mn-qpy** is neutral, whereas **Mn-dmqpy** is a divalent cation due to introduction of the quaternized dmqpy^2+^ ligand. As described above, incorporating both Br^−^ and PF_6_^−^ as counterions led to the more facile formation of single crystals suitable for X-ray diffraction. The structural analysis data suggest that the two kinds of counterions (i.e., PF_6_^−^ and Br^−^) were present in equal quantities. Actually, from the EDX analysis of the crystals, the Mn/Br/P molar ratio could be estimated to be nearly 1:2:1, which strongly supports the results of the structural analysis. The dihedral angles between the free pyridine (or pyridinium) rings and the coordinated pyridine rings were found to be small (mean values of 10.08 and 14.4°, respectively; Table 2) but distinct from those of the optimized structures (30.58 and 30.88° for **Mn-qpy** and 35.56 and 35.65° for **Mn-dmqpy**). This difference can be ascribed in part to some packing effects in the crystal. In particular, **Mn-dmqpy** constructs essential quasi-coplanarities due to multiple hydrogen bonds (C–H^…^Br) between the counterion and the free pyridinium rings (Figure 6 and Table S2), in addition to exhibiting intermolecular π-π stacking (3.704(3) Å for the centroid-centroid distance).

### 2.3. Redox Properties of the Tricarbonylmanganese Complexes

#### 2.3.1. Phenanthroline-Based Complexes: Interconversion between Quinone and Catechol Units

**Figure 7:**
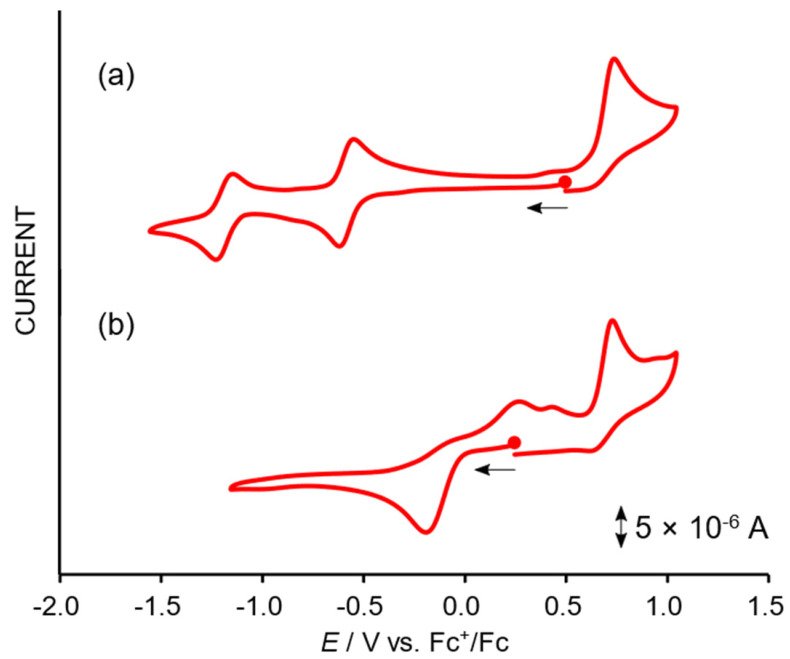
Cyclic voltammograms of [MnBr(CO)_3_(dpq)] in the absence and presence of H^+^ in CH_3_CN (glassy carbon WE): (**a**) 0 equiv. and (**b**) 2 equiv. of H^+^ (*v* = 0.1 V s^−1^). An aqueous solution of HClO_4_ (0.1 M) was used as the proton source.

**Scheme 3:**
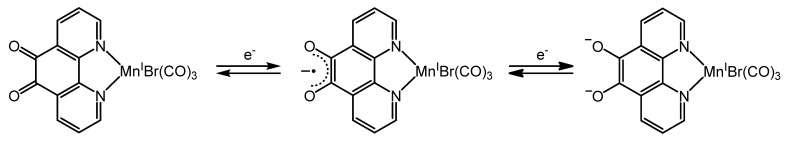
Two successive one-electron reductions of the dpq ligand under aprotic conditions.

**Figure 8:**
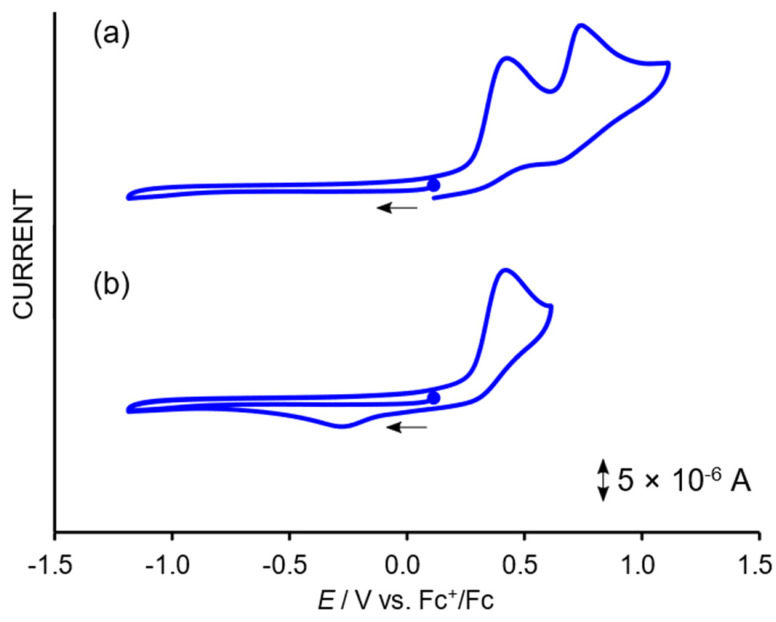
Cyclic voltammograms of [MnBr(CO)_3_(dpc)] in CH_3_CN (glassy carbon WE): (**a**) the whole scan and (**b**) the anodic scan reversed after the first oxidation peak (*v* = 0.1 Vs^−1^).

In the reference **Mn-phen**, two irreversible one-electron reductions and an oxidation wave were observed. These reductions rapidly led to the loss of bromide, followed by dimerization on the timescale of the cyclic voltammetry (CV) measurements [13]. In contrast, the electrochemical behaviors of **Mn-dpq** and **Mn-dpc** differed significantly. Initially, CV measurements were performed on **Mn-dpq** containing the quinone unit; either acetonitrile or a dilute aqueous HClO_4_ solution as a proton source were used as the solvent for carrying out the CV measurements. As previously reported, an irreversible oxidation wave (*E*_pa_ = 0.74 V) attributed to Mn^II/I^ was observed, in addition to two reversible redox pairs (*E*_1/2_ = −0.58 and −1.19 V) under aprotic conditions (Table 3 and Figure 7a) [13]. These successive waves were attributed to the stepwise electron transfers between quinone and the catecholate dianion via the semiquinone anion radical (Scheme 3) [13,35]. These potentials are shifted towards more positive values (210–350 mV) than those of the free ligand (dpq) due to complexation with the {Mn(CO)_3_}^+^ moiety [13]. When two equivalents of protons were added to the solution, a new reduction wave, in which two sets of redox pairs were united, appeared at a more positive potential (−0.2 V), and small oxidation waves were observable on the anodic scan after the reduction (i.e., at 0.27 and 0.43 V) (Figure 7b) [36]. This result suggests that proton-coupled electron transfer (PCET) occurs upon the addition of protons to generate the corresponding catechol unit directly by 2e^−^/2H^+^ transfers, as shown in Scheme 1a. On the other hand, in the CV of **Mn-dpc**, which contains the catechol unit, two oxidation waves were observed at 0.43 and 0.74 V (Figure 8a) [37]. In general, the first oxidation wave was assigned to oxidation of the catechol site [38], and our DFT calculation results also supported this assumption. When the anodic scan was immediately reversed after the first peak potential, a coupled reduction wave was observed at −0.28 V (Figure 8b). Since this reduction potential almost coincides with that of the quinone moiety during PCET (Figure 7b), it appears that **Mn-dpq** and **Mn-dpc** can electrochemically interconvert between one another via the PCET process.

**Figure 9:**
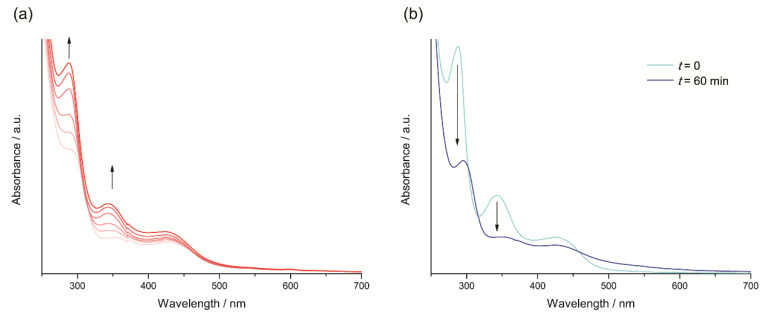
(**a**) UV-visible spectroelectrochemistry (UV/vis-SEC) data for [MnBr(CO)_3_(dpq)] in the CH_3_CN/2 equiv. of H^+^ at −0.6 V. (**b**) Spectral changes of [MnBr(CO)_3_(dpc)] in CH_3_CN under air.

**Scheme 4:**
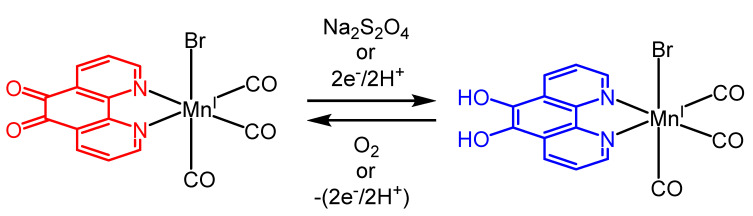
Schematic representation of the chemical and electrochemical interconversions between [MnBr(CO)_3_(dpq)] and [MnBr(CO)_3_(dpc)].

To examine the reversible structural changes induced by electrochemical and chemical reactions, the interconversion between the two complexes was monitored by absorption spectroscopy. The spectral changes of **Mn-dpq** observed during the course of the electrolysis at −0.6 V in the presence of protons are shown in Figure 9a. Upon application of the potential, the new bands at 288 and 343 nm gradually increased in intensity, and the final spectrum obtained matched that of the corresponding **Mn-dpc**. This supports the results of the CV experiments discussed above (Figure 7b), i.e., the quinone unit was completely converted to catechol through reduction in the presence of protons. In contrast, when the acetonitrile solution of **Mn-dpc** was allowed to stand for 1 h in the air, the spectrum matched that of **Mn-dpq** (Figure 9b), indicating that the catechol unit of **Mn-dpc** returned to the corresponding quinone by oxidation under air. Considering that **Mn-dpc** can be prepared by the chemical reduction of **Mn-dpq**, both the chemical and electrochemical interconversions shown in Scheme 4 are possible between **Mn-dpq** and **Mn-dpc**.

#### 2.3.2. Bipyridine-Based Complexes

The redox properties of the reference **Mn-bpy** are also similar to those of the **Mn-phen** species described above [4,13,39]. Unclear redox waves were observed in **Mn-qpy** owing to its poor solubility in almost all organic solvents (Table 3 and Figure S2). Since an oxidation wave of the species generated by an EC reaction (electron transfer followed by a homogeneous chemical reaction) was observed at −0.42 V in the CV of **Mn-qpy**, the formation of a Mn-Mn dimer upon reduction is expected, as in the case of **Mn-bpy** [40]. On the other hand, four successive reduction waves were observed in the CV of **Mn-dmqpy** (Table 3 and Figure S3). More specifically, the first reduction wave was observed at −1.14 V, which was a more positive potential than those observed for dmqpy^2+^ (−1.45 V) and **Mn-bpy** (−1.73 V) under the same conditions. DFT calculations suggested that this is attributable to the reduction of the ligand moiety (dmqpy^2+^). Since the reduction properties of **Mn-dmqpy** are similar to those of a ruthenium complex containing the dmqpy^2+^ ligand [15], the quaternization of the bpy-based ligand facilitates electron transfer. In addition, considering that no oxidation wave was observed at approximately −0.4 V during the reduction, it appeared **Mn-dmqpy** does not undergo dimer formation. This result confirmed that the reduction properties of the bipyridine-based complexes are also affected considerably by the differences in the redox-active unit.

## 3. Materials and Methods

### 3.1. General Remarks

All chemicals were used without further purification unless otherwise stated. All solvents purchased for organic synthesis were anhydrous and were used without further purification. Acetonitrile for the electrochemical measurements was purified using a Glass Contour Ultimate Solvent System (Laguna, CA, USA). [MnBr(CO)_5_] was purchased from Sigma-Aldrich (Tokyo, Japan). The ligands (dpq, qpy, and dmqpy^2+^) and the complexes ([MnBr(CO)_3_(dpq)], [MnBr(CO)_3_(phen)], and [MnBr(CO)_3_(bpy)]) were synthesized according to previously reported procedures [13,23,24,41,42]. The formation of these compounds was confirmed by MS, ^1^H-NMR, and IR spectral data. All manganese(I) complexes were handled and stored in the dark to minimize exposure to light.

Elemental analysis data were obtained on a PerkinElmer 2400II series CHN analyzer (Yokohama, Japan). IR spectra were obtained using a JASCO FT-IR 4100 spectrometer (Tokyo, Japan). Electrospray ionization mass spectrometry (ESI-MS) data were obtained using a Bruker Daltonics micrOTOF spectrometer (Yokohama, Japan). UV-visible spectra were obtained on a JASCO V-560 spectrophotometer (Tokyo, Japan). ^1^H and ^13^C{^1^H}-NMR spectra were acquired on a JEOL JMN-AL300 spectrometer (Tokyo, Japan) operating at ^1^H and ^13^C frequencies of 300 and 75.5 MHz, respectively. EDX analyses were performed using a JEOL JSM-6380LANV/EX37001 system (Tokyo, Japan). Electrochemical measurements were performed on an electrochemical analyzer (ALS/CHI model 660E, Tokyo, Japan) with a solution of the complex in acetonitrile (1 mM of **Mn-dpq** and **Mn-dpc**) or in DMF (1 mM of **Mn-dmqpy** and 0.5 mM of **Mn-qpy**) and with *n*-Bu_4_NClO_4_ (0.1 M) as a supporting electrolyte in a cell consisting of a glassy carbon or a platinum working electrode (*ϕ* = 1.6 mm), a Pt wire counter electrode, and Ag/AgNO_3_ (0.01 M) as the reference electrode. All potentials were reported in volts vs. the ferrocenium/ferrocene couple (Fc^+^/Fc) under Ar at 25 °C. UV/vis-SEC was carried out using a homemade thin-layer electrode cell with a Pt mesh working electrode sandwiched between two glass sides of an optical quartz cell (path length 0.5 mm), where an Ag/Ag^+^ reference electrode was separated from the working compartment by Vycor glass. A Hokuto Denko HAB-151 potentiostat (Tokyo, Japan) was employed to control the cell potential. Thin-layer bulk electrolysis was monitored by UV-vis spectroscopy at regular time intervals. DFT calculations were performed using the quantum computation software *Gaussian 09* [43]. The geometries of the complexes were fully optimized using a restricted DFT method employing the B3LYP function [44,45] at the 6-31G(d) level of theory with a LanL2DZ basis set [46,47,48]. The solvent effect of acetonitrile or DMF was evaluated using an implicit solvent model and a polarizable continuum model. Vibrational analyses were performed at the same calculation level employed for geometry optimization.

### 3.2. Synthesis of the Complexes

#### 3.2.1. Synthesis of fac-[MnBr(CO)_3_(dpc)]

A Schlenk flask was charged with [MnBr(CO)_3_(dpq)] (25 mg, 59 µmol), HBr aq. (1 M, 150 µL), Na_2_S_2_O_4_ aq. (1 M, 90 µL), and degassed CH_3_CN (3 mL) at 0 °C. The resulting mixture was vigorously stirred at ambient temperature under N_2_ for 30 min. After the addition of CH_3_CN (5 mL) to the solution, the reaction mixture was allowed to stand at −20 °C overnight. After this time, some insoluble matter was removed by filtration, and the volume of the filtrate was reduced to 1 mL using a rotary evaporator. Following the addition of diethyl ether (30 mL) to the solution, it was allowed to stand at −20 °C to yield [MnBr(CO)_3_(dpc)] as a yellow precipitate. The product was collected by filtration, washed with diethyl ether, and dried in vacuo. Yield: 20 mg (79%) [49]. IR (KBr): 2032, 1961, 1938 cm^−1^ (νCO). ^1^H-NMR (CD_3_CN): δ 9.36 (dd, *J* = 4.8, 1.5 Hz, 2H), 8.78 (dd, *J* = 8.4, 1.5 Hz, 2H), 7.84 (dd, *J* = 8.4, 5.1 Hz, 2H). All attempts to obtain ^13^C{^1^H} NMR data for the compound were unsuccessful due to its instability in solution (see Section 2.3.1).

#### 3.2.2. Synthesis of fac-[MnBr(CO)_3_(qpy)] and fac-[MnBr(CO)_3_(dmqpy)](PF_6_)_2_

[MnBr(CO)_5_] (24 mg, 88 µmol) and qpy (22 mg, 68 µmol) were dissolved in MeOH (20 mL), and the reaction mixture was stirred at ambient temperature for 24 h. After this time, the solvent was evaporated to a volume of 4 mL under reduced pressure, an excess of diethyl ether (20 mL) was added, and the resulting solution was allowed to stand at 4 °C overnight. The obtained precipitate was collected by filtration, washed with diethyl ether, and then dried under vacuum (30 mg, 86%). Anal. Calcd for [MnBr(CO)_3_(qpy)]·0.5H_2_O: C_23_H_15_N_4_O_3.5_BrMn: C, 51.32; H, 2.81; N, 10.41. Found: C, 51.35; H, 2.98; N, 10.69. IR (KBr): 2027, 1936, 1914 cm^−1^ (νCO). ^1^H-NMR (DMSO-*d*_6_): δ 9.31 (d, *J* = 5.7 Hz, 2H), 9.20 (s, 2H), 8.84 (s, 4H), 8.15 (d, *J* = 6.9 Hz, 2H), 8.06 (s, 4H). ^13^C{^1^H}-NMR (DMSO-*d*_6_): δ 155.90, 154.05, 147.44, 142.65, 130.22, 128.32, 126.95, 124.31.

A similar reaction between [MnBr(CO)_5_] (26 mg, 92 µmol) and dmqpy(PF_6_)_2_ (22 mg, 34 µmol) in acetone (20 mL) afforded [MnBr(CO)_3_(dmqpy)](PF_6_)_2_. Yield: 24 mg (80%). Anal. Calcd for [MnBr(CO)_3_(dmqpy)](PF_6_)_2_: C_25_H_20_N_4_O_3_F_12_P_2_BrMn: C, 33.92; H, 2.73; N, 6.33. Found: C, 33.56; H, 2.53; N, 6.10. ESI-MS (CH_3_CN): *m/z* 280.0 ([M]^2+^). IR (KBr): 2029, 1944, 1927 cm^−1^ (νCO). ^1^H-NMR (DMSO-*d*_6_): δ 9.50 (d, *J* = 5.7 Hz, 2H), 9.37 (s, 2H), 9.28 (d, *J* = 6.3 Hz, 4H), 8.81 (d, *J* = 6.0 Hz, 4H), 8.35 (d, *J* = 6.3 Hz, 4H), 4.44 (s, 6H). ^13^C{^1^H}-NMR (DMSO-*d*_6_): δ 157.47, 155.80, 152.35, 147.89, 145.10, 127.19, 125.81, 123.75, 49.45.

### 3.3. X-ray Crystallographic Analyses

Single crystals of [MnBr(CO)_3_(dpq)]·(CH_3_)_2_CO (orange plate-like crystals), [MnBr(CO)_3_(dpc)] (yellow block-like crystals), [MnBr(CO)_3_(qpy)] (orange block-like crystals), and [MnBr(CO)_3_(dmqpy)]Br(PF_6_) (reddish-orange plate-like crystals) were obtained by the vapor diffusion of *n*-hexane into an acetone solution of the complex. Crystal structure determination and refinement data for the complexes are given in Figure 3, Figure 4, Figure 5 and Figure 6, Table 2, Table 4, and Supplementary Materials Figure S1, Table S1 and Table S2. The occupancies of the counter-anions (Br^−^ and PF_6_^−^) in [MnBr(CO)_3_(dmqpy)]Br(PF_6_) were 1:1. We confirmed the CIF data by using the *checkCIF/PLATON* tests [50]. CCDC-2039786–2039789 contains the supplementary crystallographic data for this paper.

## 4. Conclusions

We herein demonstrated that various functions can be imparted onto conventional manganese complexes by introducing redox-active units into typical bidentate polypyridyl ligands. In particular, metal complexes containing quinone/catechol units that cause a reversible proton-coupled electron transfer can interconvert by redox reactions, thereby leading to a variety of applications, such as the construction of a reversible molecular switch based on an external stimulus. Our new manganese compounds will open up new pathways, not only for systematic research into the molecular structures and catalytic properties of manganese compounds but, also, for the creation of new functions for manganese complexes. Further studies are underway to explore a manganese-catalyzed redox system for the reduction of carbon dioxide to some high value-added organics, and the results will be presented in due course.

## Figures and Tables

**Table 1:** Spectral data for the Mn(I) complexes. IR: infrared.

Technique	Mn-dpq	Mn-dpc	Mn-qpy	Mn-dmqpy
IR (νCO/cm^−1^)	2033	2032	2027	2029
	1944 1928 1698	1961 1938	1936 1914	1944 1927
UV-vis (λ_max_/nm)	427 (2.4)	425 (1.9)	427 (1.4)	468 (4.1)
(ε/×10^3^ M^−1^cm^−1^)	352 (2.9) 294 (9.0)	343 (4.1) 288 (12)	297 (11)	319 (17)

**Table 2:** Selected bond lengths (Å) and angles (°) for the Mn(I) complexes.

Parameter	Mn-dpq		Mn-dpc		Mn-qpy		Mn-dmqpy	
	Expt.	Calc.	Expt.	Calc.	Expt.	Calc.	Expt.	Calc.
Mn1-C1	1.799(5)	1.801	1.812(2)	1.797	1.813(2)	1.797	1.806(8)	1.801
Mn1-C2	1.817(7)	1.815	1.804(2)	1.813	1.8123(19)	1.814	1.829(8)	1.817
Mn1-C3	1.810(7)	1.815	1.809(2)	1.813	1.8054(17)	1.815	1.834(12)	1.817
Mn1-N1	2.054(4)	2.077	2.0452(17)	2.080	2.0344(15)	2.066	2.051(6)	2.063
Mn1-N2	2.049(4)	2.077	2.0614(19)	2.080	2.0369(12)	2.065	2.045(7)	2.063
Mn1-Br1	2.5385(7)	2.599	2.5628(10)	2.610	2.5250(4)	2.610	2.5617(12)	2.600
C1-O1	1.133(7)	1.156	1.121(3)	1.157	1.120(3)	1.156	1.124(11)	1.155
C2-O2	1.145(9)	1.154	1.141(3)	1.155	1.142(2)	1.155	1.140(11)	1.154
C3-O3	1.151(8)	1.154	1.143(3)	1.155	1.145(2)	1.155	1.091(16)	1.154
C9-O4	1.212(6)	1.217	1.362(2)	1.353				
C10-O5	1.220(7)	1.217	1.367(2)	1.353				
Mn1-C1-O1	177.5(6)	179.9	178.53(19)	179.9	176.81(17)	179.9	173.1(9)	180.0
Mn1-C2-O2	177.5(6)	178.9	177.09(18)	178.9	176.06(15)	178.9	178.6(7)	178.8
Mn1-C3-O3	176.5(5)	178.8	177.56(18)	178.9	177.8(2)	178.9	176.5(9)	178.8
R1/R3 ^1^					15.52(7)	30.58	16.8(3)	35.56
R2/R4 ^2^					4.63(7)	30.88	12.0(3)	35.65

^1^ Dihedral angle between Ring 1 (N1-pyridine) and Ring 3 (N3-pyridine). ^2^ Dihedral angle between Ring 2 (N2-pyridine) and Ring 4 (N4-pyridine).

**Table 3:** Electrochemical data in aprotic media for the Mn(I) complexes.

Reaction (V vs. Fc^+^/Fc)	Mn-dpq	Mn-dpc	Mn-qpy	Mn-dmqpy
Reductions (Δ*E*)	−0.58 ^1^ (0.07)	−0.28 ^3,4^	−1.48 ^3^	−1.14 ^1^ (0.06)
	−1.19 ^1^ (0.08)		−1.56 ^3^	−1.36 ^3^
				−1.78 ^1^ (0.08)
				−2.20 ^1^ (0.09)
Oxidations	0.74 ^2^	0.43 ^2^	−1.26 ^2^	−1.34 ^2^
		0.74 ^2^	−0.42 ^2,4^	0.34 ^2,5^
			0.35 ^2,4^	

^1^*E*_1/2_ value. ^2^*E*_pa_ value. ^3^*E*_pc_ value. ^4^ The waves appear by the corresponding EC reaction. ^5^ This wave is assignable to the oxidation of a free Br^−^, which is partially contained as a counter ion.

**Table 4:** Crystallographic data for the Mn(I) complexes.

Parameter	Mn-dpq [MnBr(CO)_3_(dpq)](CH_3_)_2_CO	Mn-dpc [MnBr(CO)_3_(dpc)]	Mn-qpy [MnBr(CO)_3_(qpy)]	Mn-dmqpy [MnBr(CO)_3_(dmqpy)]BrPF_6_
Formula	C_18_H_12_BrMnN_2_O_6_	C_15_H_8_BrMnN_2_O_5_	C_23_H_14_BrMnN_4_O_3_	C_25_H_20_Br_2_F_6_MnN_4_O_3_P
Formula weight	487.14	431.08	529.23	784.17
Temperature (K)	93	93	93	93
Crystal system	monoclinic	triclinic	monoclinic	triclinic
Space group	*P*2_1_/*c*	*P*-1	*P*2_1_/*c*	*P*-1
*a* (Å)	20.3113(11)	7.077(3)	11.8530(2)	6.4688(3)
*b* (Å)	6.9096(3)	9.759(4)	18.0938(3)	12.4896(6)
*c* (Å)	14.1250(7)	11.600(5)	10.53500(19)	18.8132(10)
*α* (°)	90	91.966(4)	90	72.4654(14)
*β* (°)	108.8940(14)	107.572(3)	111.2740(7)	89.3644(14)
*γ* (°)	90	104.311(5)	90	82.1867(13)
*V* (Å^3^)	1875.54(16)	734.9(6)	2105.43(7)	1435.18(12)
*Z*	4	2	4	2
Calcd density (g/cm^3^)	1.725	1.948	1.669	1.814
*μ* (Mo *Kα*) (mm^−1^)	2.879	3.655	2.564	3.383
No. unique reflns	18452	7485	21537	14809
No. obsd reflns	4280	3303	4815	6510
Refinement method	Full-matrix least squares on *F*^2^			
Parameters	255	217	289	379
*R* (*I* > 2*σ*(*I*)) ^1^	0.0572	0.0247	0.0254	0.0776
*wR* (all data) ^2^	0.1480	0.0667	0.0642	0.2171
*S*	1.042	1.065	1.072	1.025

^1^*R* = Σ(||*F*_o_| − |*F*_c_||)/Σ|*F*_o_|; ^2^ *wR* = {Σ*_w_*(*F*_o_^2^ − *F*_c_^2^)^2^/Σ*_w_*(*F*_o_^2^)^2^}^1/2^.

## Supplementary Materials

Figure S1: A dimer formation caused by intermolecular hydrogen bonds and π-π stacking in the crystal packing of **Mn-dpc**. Figure S2: Cyclic voltammogram of **Mn-qpy** in DMF (*v* = 0.1 V s^–1^, *c* = 0.5 mM). Figure S3: Cyclic voltammogram of **Mn-dmqpy** in DMF (*v* = 0.1 V s^–1^, *c* = 1 mM). Table S1: Hydrogen-bond geometry (Å, °) for **Mn-dpq** and **Mn-dpc**. Table S2: Hydrogen-bond geometry (Å, °) for **Mn-qpy** and **Mn-dmqpy**.
