# Supplementary material for: Effects of Chemically-Modified Polypyridyl Ligands on the Structural and Redox Properties of Tricarbonylmanganese(I) Complexes
Source: Molecules. 2020 Dec 14;25(24):5921. doi: 10.3390/molecules25245921 (PMC7765023; doi:10.3390/molecules25245921)
Supplement: Supplementary file 1 [file molecules-25-05921-s001.pdf]

molecules

MDPI

Article

Effects of Chemically-Modified Polypyridyl Ligands on the Structural and Redox Properties of Tricarbonylmanganese(I) Complexes

Takatoshi Kanno [1], Tsugiko Takase [2] and Dai Oyama [2,*]

[1] Graduate School of Science and Engineering, Fukushima University, 1 Kanayagawa, Fukushima 960-1296, Japan; s1970013@ipc.fukushima-u.ac.jp

[2] Department of Natural Sciences and Informatics, Fukushima University, 1 Kanayagawa, Fukushima 960-1296, Japan; ttakase@sss.fukushima-u.ac.jp

* Correspondence: daio@sss.fukushima-u.ac.jp; Tel.: +81-24-548-8199

Academic Editor: Rudy J. Richardson

Table S1. Hydrogen-bond geometry (Å, °) for **Mn-dpq** and **Mn-dpc**.

<Mn-dpq>

D–H· · ·A	*D*–H	H· · ·A	*D*· · ·A	*D*–H· · ·A
C5–H2· · ·Br1^{i}	0.95	2.93	3.681(5)	137
C13–H4· · ·Br1^{ii}	0.95	2.85	3.536(5)	130
C18–H10· · ·O4^{iii}	0.98	2.60	3.399(7)	139
C18–H12· · ·O4^{iv}	0.98	2.38	3.333(7)	163

Symmetry codes: (i) $x, 1+y, z$; (ii) $x, -\frac{1}{2}-y, -\frac{1}{2}+z$; (iii) $2-x, -y, 1-z$; (iv) $x, \frac{1}{2}-y, \frac{1}{2}+z$

<Mn-dpc>

D–H· · ·A	*D*–H	H· · ·A	*D*· · ·A	*D*–H· · ·A
O4–H1· · ·O5	0.84	2.56	2.949(2)	110
O4–H1· · ·Br1^{i}	0.84	2.46	3.2601(19)	159
O5–H2· · ·O4	0.84	2.56	2.949(2)	110
O5–H2· · ·Br1^{i}	0.84	2.50	3.304(2)	162
C6–H5· · ·O2^{ii}	0.95	2.36	3.080(3)	132

Symmetry codes: (i) $1-x, -y, 2-z$; (ii) $-1+x, -1+y, z$

Table S2. Hydrogen-bond geometry (Å, °) for **Mn-qpy** and **Mn-dmqpy**

<Mn-qpy>

D–H· · ·A	*D*–H	H· · ·A	*D*· · ·A	*D*–H· · ·A
C16–H8· · ·O1^{i}	0.95	2.60	3.399(3)	142
C18–H10· · ·Br1^{ii}	0.95	2.92	3.7693(19)	149
C21–H12· · ·O2^{iii}	0.95	2.53	3.461(3)	168

Symmetry codes: (i) $1-x, -\frac{1}{2}+y, \frac{1}{2}-z$; (ii) $2-x, 2-y, 1-z$; (iii) $2-x, -\frac{1}{2}+y, \frac{3}{2}-z$

<Mn-dmqpy>

D–H· · ·A	*D*–H	H· · ·A	*D*· · ·A	*D*–H· · ·A
C7–H3· · ·Br2	0.95	2.86	3.810(8)	175
C10–H4· · ·Br2	0.95	2.89	3.843(7)	177
C13–H6· · ·Br1^{i}	0.95	2.92	3.573(7)	127
C15–H7· · ·F5^{ii}	0.95	2.54	3.181(16)	125
C18–H10· · ·Br2	0.95	2.64	3.492(11)	150
C19–H12· · ·F4^{iii}	0.98	2.51	3.278(13)	135
C19–H13· · ·F1^{iv}	0.98	2.51	3.259(14)	133
C21–H14· · ·Br2	0.95	2.68	3.567(9)	156
C25–H20· · ·O1^{v}	0.98	2.21	3.088(12)	148

Symmetry codes: (i) $-1+x, y, z$; (ii) $1+x, y, z$; (iii) $2-x, 1-y, -z$; (iv) $3-x, 1-y, -z$; (v) $x, -1+y, z$

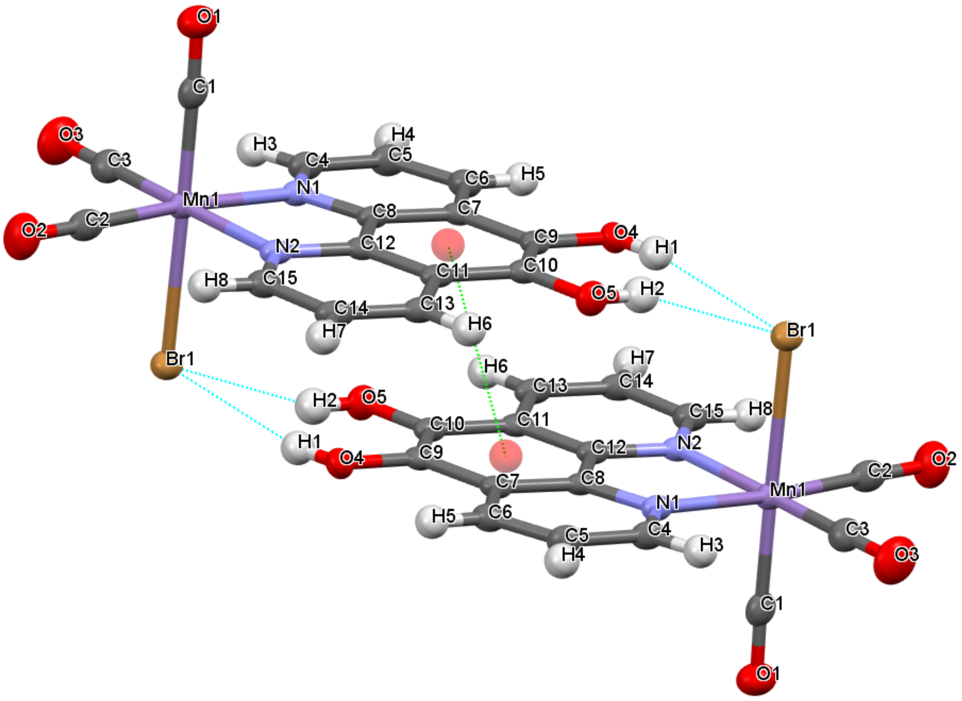

Figure S1. A dimer formation caused by intermolecular hydrogen bonds and π-π stacking in the crystal packing of **Mn-dpc**.

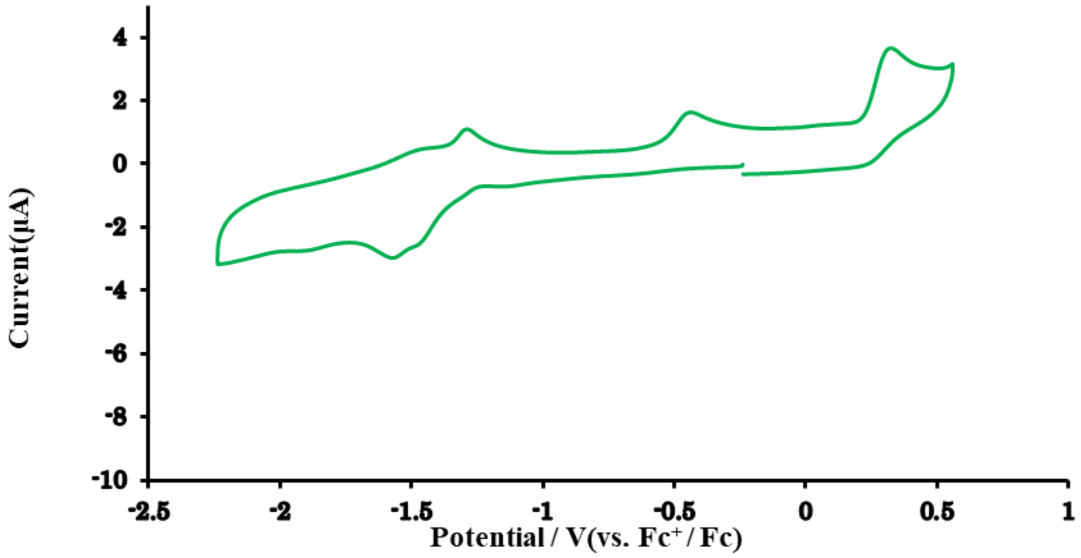

Figure S2. Cyclic voltammogram of **Mn-qpy** in DMF (v = 0.1 V s^{-1}, c = 0.5 mM).

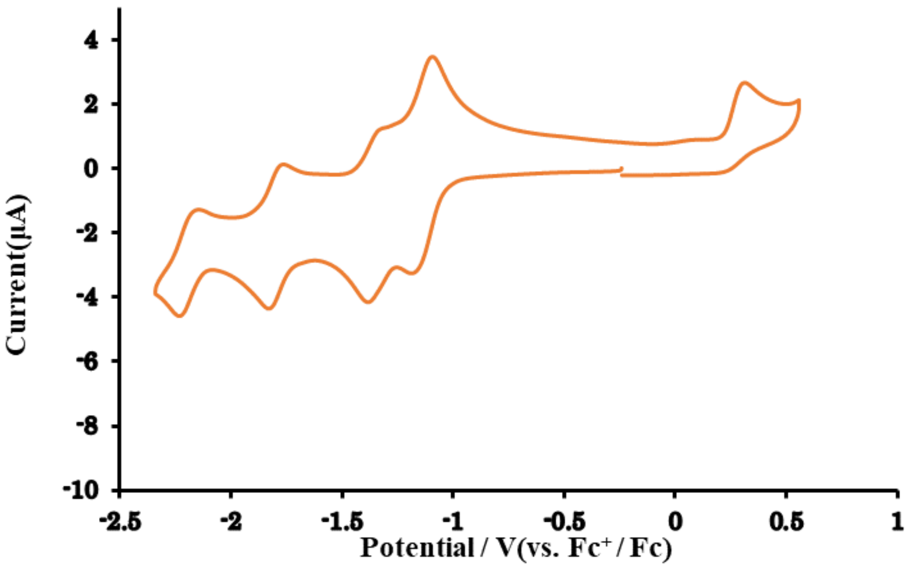

Figure S3. Cyclic voltammogram of **Mn-dmqpy** in DMF (v = 0.1 V s^{-1}, c = 1 mM).
